# Scientific Rationale for Combined Immunotherapy with PD-1/PD-L1 Antibodies and VEGF Inhibitors in Advanced Hepatocellular Carcinoma

**DOI:** 10.3390/cancers12051089

**Published:** 2020-04-27

**Authors:** Masatoshi Kudo

**Affiliations:** Department of Gastroenterology and Hepatology, Kindai University Faculty of Medicine, Osaka-Sayama 589-8511, Japan; m-kudo@med.kindai.ac.jp; Tel.: +81-72-366-0221 (ext. 3149); Fax: +81-72-367-2880

**Keywords:** hepatocellular carcinoma, immune checkpoint inhibitor, PD-1 antibody, PD-L1 antibody, anti-VEGF inhibitor

## Abstract

A successful phase III trial for the combination of atezolizumab and bevacizumab (the IMbrave150 trial) in advanced hepatocellular carcinoma has recently been reported. This is groundbreaking because nivolumab and pembrolizumab, both programmed cell death-1 (PD-1) antibodies, have failed to show efficacy as first- and second-line therapeutics, respectively, in phase III clinical trials. Immunotherapy with a combination of atezolizumab and bevacizumab resulted in better survival than treatment with sorafenib for the first time since sorafenib was approved in 2007. The high efficacy of the combination of PD-1/programmed death ligand 1 (PD-L1) and vascular endothelial growth factor (VEGF) antibodies is not only due to their additive effects on tumor growth, but also to their reprogramming of the immunosuppressive microenvironment into an immunostimulatory microenvironment. These results were confirmed in a phase Ib trial that showed significantly longer progression-free survival in the atezolizumab plus bevacizumab group than in patients that received atezolizumab alone. These results demonstrate that immunotherapy with a combination of PD-1/PD-L1 and VEGF inhibitors is effective and may result in a reprogramming of the tumor microenvironment. The results of an ongoing phase III trial of a PD-1 antibody in combination with the VEGF receptor tyrosine kinase inhibitor (TKI) are highly anticipated.

## 1. Introduction

At the European Society for Medical Oncology (ESMO) Asia in November 2019, the positive results of the IMbrave150 study, a trial which compared the effects of the combination of atezolizumab and bevacizumab with those of sorafenib [1], drew attention to the possibility of immunotherapy with a combination of programmed cell death-1 (PD-1)/programmed death ligand 1 (PD-L1) and vascular endothelial growth factor (VEGF) inhibitors. This review outlines the scientific rationale for the therapeutic combination of PD-1/PD-L1 and VEGF antibodies, proof-of-concept results of the phase Ib trial, and results of other phase Ib trials for similar combination strategies.

## 2. The Rationale Underlying the Combination of PD-1/PD-L1 and VEGF Inhibitors

**Figure 1:**
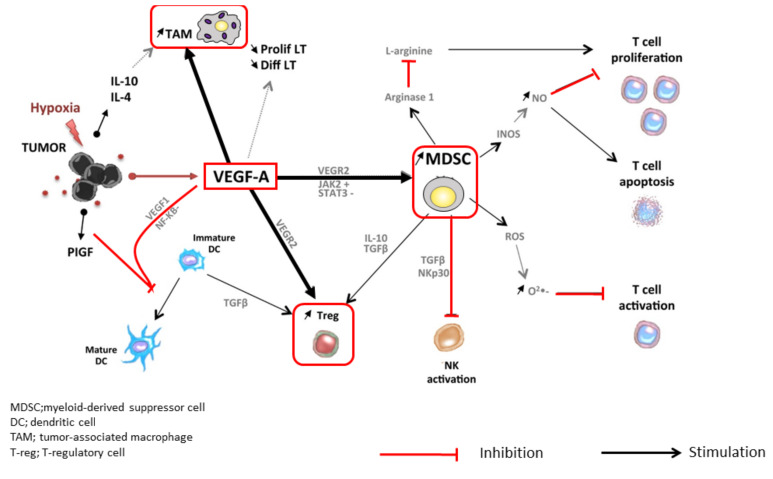
Immune suppressive microenvironment induced by VEGF (modified from ref. [4] with permission).

**Figure 2:**
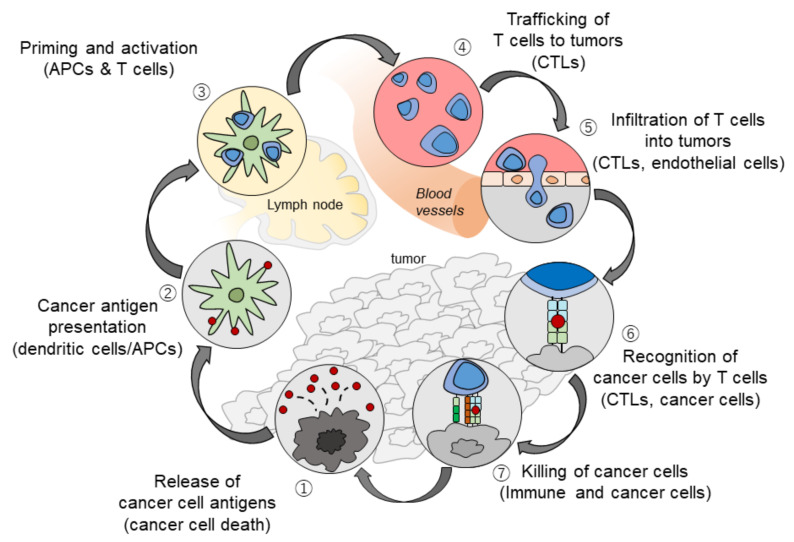
The Cancer-Immunity Cycle (modified from ref. [5] with permission).

At tumor sites, VEGF released by hypoxic cancer cells and vascular endothelial cells promotes tumor growth, invasion, and metastasis by increasing neovascularization [2]. Simultaneously, VEGF enhances the mobilization and proliferation of various cells, including regulatory T cells (Tregs), and the release of immunosuppressive cytokines [2,3]. It also enhances the mobilization of tumor-associated macrophages (TAMs) and their polarization to an M2 phenotype. Tregs and TAMs promote tumor growth through the release of VEGF and angiopoietin-2, among other mechanisms [4]. VEGF can also activate myeloid-derived suppressor cells (MDSCs), which in turn release more VEGF [4]. Furthermore, VEGF inhibits dendritic cell maturation and antigen presentation in the priming phase. Thus, VEGF reduces the proliferation and activation of naive CD8+ cells by suppressing dendritic cell activity even in the presence of neoantigens [4] (Figure 1). VEGF-induced Tregs, TAMs, and MDSCs reduce the proliferation and function of CD8+ cells. VEGF also prevents antigen-activated CD8+ cells from infiltrating the tumor tissue through its effects on tumor angiogenesis. In addition, VEGF creates a microenvironment that inhibits the function of T cells in the tumor during the effector phase of the immune response [4]. Furthermore, immunosuppressive cells (Tregs, TAMs, and MDSCs) promote immune escape by releasing immunosuppressive cytokines, including interleukin (IL)-10 and transforming growth factor beta (TGF-β), and by inhibiting dendritic cell maturation and activation, NK cell activation, and T cell activation and proliferation [2,3,4,5,6,7,8,9,10,11,12,13,14,15,16,17,18,19,20,21,22,23,24,25] (Figure 1). The cancer immunity cycle begins with the uptake and presentation of neoantigens released from necrotic tumor cells by dendritic cells. This is followed by seven steps: (1) tumor antigen release, (2) tumor antigen uptake and presentation by dendritic cells, (3) T cell priming and activation, (4) T cell migration to the tumor, (5) T cell invasion of the tumor, (6) cancer cell recognition by T cells, and (7) attack on tumor cells by T cells, which leads to cancer cell death and release of additional tumor antigens [5] (Figure 2). VEGF promotes immune escape at almost every step of the cancer immunity cycle [6,7,8,9]. Furthermore, hepatic interstitial cells such as Kupffer cells, liver endothelial cells, and hepatic stellate cells are involved in maintaining immune tolerance in the healthy liver and may contribute to the immunosuppressive microenvironment in hepatocellular carcinoma [26].

**Figure 3:**
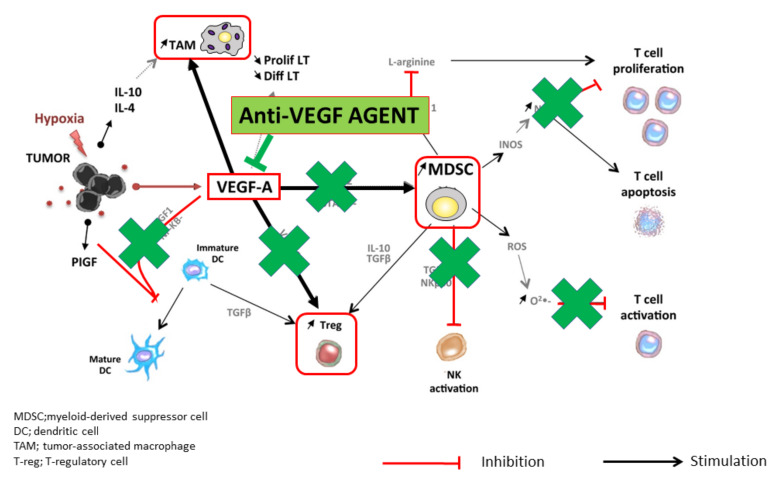
Anti-VEGF antibody reprograms the tumor microenvironment from immune suppressive to immune permissive (modified from ref. [4] with permission).

**Figure 4:**
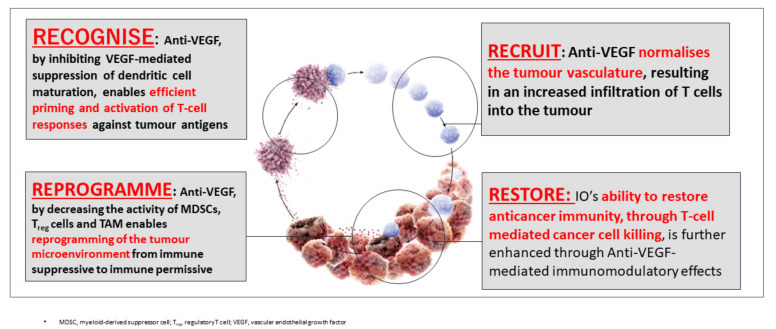
Scientific rationale of Immune-checkpoint Inhibitors plus Anti-VEGF: 4 Roles of anti-VEGF inhibitors in Cancer Immunity cycle, Recognise, Recruitment, Reprogramme, and Restore (original Figure).

The administration of molecular targeted drugs that inhibit VEGF activity, such as multi-kinase inhibitors that inhibit VEGF receptors, leads to an increase in antigen presentation by dendritic cells [8]. These drugs also promote T cell activation in the priming phase [8] and improve the migration of T cells from the lymph nodes to the tumor site by normalizing the tumor vasculature [15]. In addition, these drugs have been found to suppress the generation of Tregs, TAMs, and MDSCs at the tumor site, and to negatively regulate the expression of immunosuppressive cytokines such as TGF-β and IL-10 [10]. VEGF inhibitors therefore reprogram the immunosuppressive tumor microenvironment into an immunostimulatory environment [6,8]. The administration of PD-1/PD-L1 antibodies under such conditions enhances the antitumor activity of T cells (Figure 3 and Figure 4). As described above, the combination of VEGF and PD-1/PD-L1 inhibitors promotes antitumor immunity according to the four Rs. First, a reversal of the VEGF-mediated inhibition of dendritic cell maturation results in the effective priming and activation of T cells (Recognition) [9]. Second, anti-VEGF antibodies normalize the tumor vasculature and promote the effective infiltration of T cells into the tumor (Recruitment) [15]. Third, anti-VEGF antibodies inhibit the activity of MDSCs, Tregs, and TAMs, leading to the reprogramming of the immunosuppressive microenvironment into an immunostimulatory microenvironment (Reprogramming) [6]. Fourth, PD-1/PD-L1 antibodies enhance the ability of T cells to attack tumor cells (Restoration) (Figure 3). These four Rs lead to efficient cancer immunity and tumor growth inhibition. Proteins released by the killed tumor cells are taken up by dendritic cells, and then processed into tumor antigen peptides that are presented on major histocompatibility complex (MHC) class I molecules, leading to a progression through the cancer immunity cycle and further tumor attacks [5] (Figure 2). As described above, normalization of the VEGF-suppressed tumor microenvironment with molecular targeted agents against VEGF leads to the efficient attack on tumors by activated T cells [5,6,7,8,9,10,11,12,13,14,15,16,17,18,19,20,21,22,23,24,25,27] (Figure 2 and Figure 4). In addition, non-clinical study of lenvatinib, a tyrosine kinase inhibitor (TKI), showed that the inhibition of VEGF activity reduced TAMs and Tregs in the tumor microenvironment, leading to a decrease in TGF-β and IL-10, a decreased expression of T cell exhaustion markers such as PD-1 and TIM-3, and an increased expression of immunostimulatory cytokines such as IL-12 [28,29,30,31]. These findings form the rationale for a trial of the combination of TKIs and anti-PD-1/PD-L1 antibodies.

## 3. Classification of the Tumor Microenvironment and Determination of Immunotherapeutic Strategies

**Figure 5:**
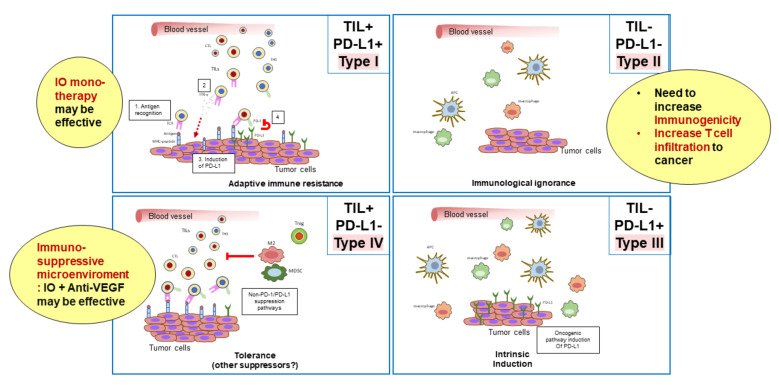
Cancer is classified into 4 types depending on immune microenvironment (TIL: CD8+ cell and PD-L1 expression) (Type I-IV) (modified from ref. [32] with permission).

Cancers are classified into four types based on the presence of tumor-infiltrating CD8+ T cells and the expression of PD-L1 [32] (Figure 5). Type I tumors contain tumor-infiltrating lymphocytes and express PD-L1. Type I cancers generally show an adequate response to monotherapy with immune checkpoint inhibitors. By contrast, type IV tumors lack PD-L1 expression, although they do contain tumor-infiltrating lymphocytes. Type IV tumors are not responsive to immune checkpoint inhibitors because the immunosuppressive tumor microenvironment inhibits the proliferation and activity of CD8+ cells in these tumors. In type I, there is an initial antitumor immune response, in which perforin, granzyme, and interferon gamma (IFN-γ) are released by activated CD8+ cells, resulting in an immune attack on the cancer cells [32]. However, IFN-γ binds to IFN-γ receptors on the cancer cell surface and upregulates the expression of PD-L1 through the Janus kinase (JAK)-signal transducer and activator of transcription (STAT) signaling pathway [31]. This leads to immune escape, whereby cancer cells evade the attack by activated CD8+ cells. Therefore, type I cancers are responsive to monotherapy with PD-1/PD-L1 antibodies. By contrast, type IV tumors do not show an initial local immune response, even though CD8+ cells are present and the tumor expression of PD-L1 is low. These tumors are never attacked by CD8+ cells because T cell activity is inhibited by the immunosuppressive microenvironment. Therefore, induction of IFN-γ and PD-L1 expression is not observed [28,32]. As expected, such cancers are not responsive to anti-PD-1/PD-L1 antibody monotherapy due to the absence of immune escape through the PD-1/PD-L1 axis. Thus, PD-1 antibody monotherapy is not predicted to be effective in cancers without PD-L1 expression, even if there are large numbers of tumor-infiltrating lymphocytes. In such tumors, anti-VEGF antibodies or inhibitors may reprogram the immunosuppressive microenvironment into an immunostimulatory microenvironment by targeting Tregs, TAMs, and MDSCs, leading to an attack by antigen-specific T cells. This, in turn, would lead to the induction of PD-L1 on the cancer cell surface by IFN-γ. In this scenario, PD-1/PD-L1 antibodies could inhibit immune escape through the PD-1/PD-L1 axis [28,32]. Therefore, this combination therapy could be effective in tumors that are unresponsive to anti-PD-1/PD-L1 monotherapy. Dramatic tumor inhibition could therefore result from the concomitant administration of PD-1/PD-L1 antibodies and VEGF antibodies or TKIs in type IV tumors (Figure 3 and Figure 4) [32]. However, in Type II and III tumors, where no tumor-infiltrating lymphocytes are present, another strategy to increase immunogenicity may be necessary. 

## 4. The Results of a Phase Ib Trial of the Combination of Atezolizumab and Bevacizumab (Clinical Trials.Gov Identifier NCT02715531)

### 4.1. The Use of the Combination of Atezolizumab (a PD-L1 Antibody) and Bevacizumab (a VEGF Antibody) in Unresectable Hepatocellular Carcinoma (Arm A) 

Arm A of NCT02715531 was a single-arm phase Ib study of the combination of atezolizumab (a PD-L1 antibody) and bevacizumab (a VEGF antibody) in unresectable hepatocellular carcinoma. Updated results from the 104 unresectable hepatocellular carcinoma patients in Arm A were presented at the annual meeting of the European Society for Medical Oncology (ESMO) in Barcelona, in the fall of 2019 [33]. Fifty-three percent of patients had macroscopic vascular invasion (MVI), of whom 88% were hepatocellular carcinoma patients with highly advanced extrahepatic spread (EHS). Although these were highly advanced cases, evaluation by an independent imaging facility (IRF) based on Response Evaluation Criteria in Solid Tumors (RECIST, version 1.1) showed an overall response rate (ORR) of 36% (95% confidence interval [CI], 26–46%). The ORR based on the modified RECIST (mRECIST) was 39%. The percentage of patients achieving a complete response (CR) based on RECIST 1.1 was 12%. Moreover, the partial response (PR) rate and disease control rate (DCR) were 24% and 71%, respectively. The median duration of response was not reached (95% CI, 11.8–not estimated [NE]). There were 20 patients (54%) with a duration of response ≥ 9 months and 11 patients (30%) with long-term responses (duration of response ≥ 12 months).

In addition, the progression-free survival (PFS) and overall survival (OS) were extremely good (PFS, 7.3 months [95% CI, 5.4–9.9]; OS, 17.1 months [95% CI, 13.8–not reached]). The result is very promising considering the fact that 53%, 88%, and 36% of patients had MVI, EHS with or without MVI, and alpha-fetoprotein (AFP) > 400 ng/mL, respectively.

### 4.2. Randomized Controlled Arm Comparing the Combination of Atezolizumab Plus Bevacizumab Versus Atezolizumab Alone (Arm F)

Arm F of the study compared PFS in unresectable hepatocellular carcinoma between the combination of atezolizumab (1200 mg) and bevacizumab (15 mg/kg) (every 3 weeks), and atezolizumab alone (1200 mg) as a first-line therapy. This was a proof-of-concept study to determine whether the favorable outcomes observed in Arm A were due to atezolizumab alone or to the combined effect of bevacizumab plus atezolizumab. Importantly, the ORR of the combination of atezolizumab and bevacizumab was slightly higher (20%) than that of atezolizumab alone (17%), which is consistent with data from other trials on the ORR of immune checkpoint inhibitors alone (about 15–18.3% [34,35,36,37,38,39]). In fact, the median PFS was 5.6 months (95% CI, 3.6–7.4) for atezolizumab plus bevacizumab, and 3.4 months (95% CI, 1.9–5.2) for atezolizumab alone. The hazard ratio was 0.55 (95% CI, 0.40–0.74; *p* = 0.0108). These data clearly showed the beneficial effect of bevacizumab on atezolizumab therapy. The PFS of atezolizumab plus bevacizumab in Arm F (5.6 months) was shorter than that in Arm A (7.3 months). However, this result may be due to the fact that the median follow-up period of Arm F was shorter (6.6 months vs. 12.4 months). With extended follow-up, the PFS in Arm F may have been equivalent to that of Arm A. In any case, the results of Arm F clearly supported the hypothesis that bevacizumab reprograms the immunosuppressive microenvironment into an immunostimulatory environment, enhancing the efficacy of atezolizumab (Figure 4).

## 5. Results of Phase Ib Studies of Other Combinations of PD-1/PD-L1 Antibodies and VEGF Inhibitors 

In addition to the trial of atezolizumab and bevacizumab described above, other studies are examining the efficacy of combined PD-1/PD-L1 and VEGF inhibition. One such study, the LEAP-002 study, is a phase III clinical trial of pembrolizumab and lenvatinib [40,41]. This trial is ongoing and the results are highly anticipated. In addition, multiple other clinical trials of immune checkpoint inhibitors and VEGF inhibitors have been completed (Table 1). The number of patients who received pembrolizumab and lenvatinib (*n* = 67) was lower than the number of patients who received atezolizumab and bevacizumab in Arm A of the phase Ib trial described above (*n* = 104). The ORR (40.3%), DCR (85.1%), PFS (9.7 months), and OS (20.4 months) of the combination of pembrolizumab and lenvatinib were higher than those of the combination of atezolizumab and bevacizumab [42]. Furthermore, the efficacy of the combination of nivolumab and lenvatinib (evaluated by an independent imaging committee based on RECIST 1.1), which was recently reported at the annual meeting of the American Society of Clinical Oncology, Gastrointestinal Cancers (ASCO GI), was higher than that of the other two combination therapies (ORR, 54.2%; DCR, 91.7%; PFS, 7.4 months; and OS, not reached) [43]. Of course, it is not adequate to compare the results of single-arm trials with different patient populations, small sample sizes, and short observation periods. However, the results are very promising. The ORR and PFS of the combination of camerelizumab and apatinib were 38.9% and 7.2 months, respectively [44]. However, there have been no updated reports on this combination. Moreover, the reported results of the combination of avelumab and axitinib [45] were slightly inferior to those of other combination therapies (ORR, 13.6%; PFS, 5.5 months; and OS, 12.7 months, based on RECIST 1.1). Therefore, at present, the most promising ongoing trial is the LEAP-002 study [40,41]. The decision whether or not to proceed to phase III trials of the combination of nivolumab and lenvatinib has currently drawn attention. In any case, the efficacy of all other combinations of anti-PD-1/PD-L1 antibodies and TKIs or anti-VEGF antibodies, except for the combination of avelumab and axitinib, is higher than that of nivolumab (a PD-1 antibody) alone (ORR, 15%; DCR, 55%; PFS, 3.7 months; and OS, 16.4 months) [34] or pembrolizumab alone (ORR, 18.3%; DCR, 62.2%; PFS, 3.0 months; OS, 13.9 months) [36]. Therefore, combined immunotherapy is expected to shift the paradigm as a first-line treatment option in advanced hepatocellular carcinoma [41,46].

## 6. Conclusions

**Figure 6:**
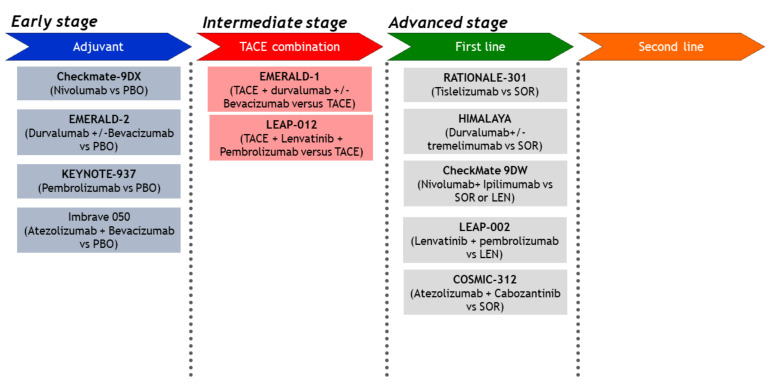
Ongoing Phase III trials in HCC (original Figure).

This article described the scientific rationale for the combination of PD-1/PD-L1 antibodies plus VEGF inhibitors, and discussed the results of a phase Ib trial of this combination. We also described the results of Arm F of a randomized phase Ib trial of the combination of atezolizumab and bevacizumab, a combination that also achieved positive results in the phase III IMbrave150 study. The results of the phase Ib trial (Arm F) and the success of the phase III IMbrave150 study suggest that the tumor microenvironment was changed by bevacizumab, enabling greater responses to the immune checkpoint blockade, as hypothesized. In addition to the improvement in PFS, in the phase III IMbrave150 study, the OS was also improved, which was an unexpected finding [1]. These results are paradigm-changing as well as practice-changing. This study suggested that the immunosuppressive tumor microenvironment was successfully reprogrammed into an immunostimulatory microenvironment that was responsive to an immune checkpoint blockade. Therefore, the promising results that have been reported with combinations of anti-PD-1/PD-L1 antibodies and VEGF inhibitors (bevacizumab or TKIs) may be due to a normalization of the tumor microenvironment. In addition to the combination of atezolizumab and bevacizumab, therapies with other combinations targeting the same pathways (Table 1), especially the combinations of penbrolizumab and lenvatinib (the LEAP-002 study) and atezolizumab and cabozantinib (the COSMIC-312 trial), are highly promising (Figure 6 and Table 2) [1,34,36,47,48,49,50,51,52,53,54,55,56,57,58,59,60,61,62,63,64,65]. Furthermore, other phase III trials of combinations with CTLA-4 inhibitors [66] (durvalumab plus tremelimumab [HIMALAYA study] and nivolumab plus ipilimumab [the CheckMate 9DW study]) are currently being conducted (Figure 1 and Table 2). In the era of combination immunotherapy, the treatment of hepatocellular carcinoma, including the proper use of molecular targeted drugs after progression on immunotherapy [67,68], has entered a period of a major paradigm shift. 

## Figures and Tables

**Table 1:** Efficacy of Immune Checkpoint Inhibitors and Combination Immunotherapy with VEGF Antibodies/Tyrosine Kinase Inhibitors in Phase 1b Trials according to RECIST 1.1.

Efficacy	Anti-PD-1 Monotherapy (Phase 3 Trial)		Anti-PD-1/PD-L1 plus TKI/Anti-VEGF (Phase 1b Trial)				
	Nivolumab [34] (*n* = 214)	Pembrolizumab [36] (*n* = 278)	Atezolizumab + bevacizumab [33] (*n* = 104)	Pembrolizumab + Lenvatinib [42] (*n* = 67)	Camrelizumab + apatinib [44] (*n* = 18)	Avelumab + axitinib [45] (*n* = 22)	Nivolumab + Lenvatinib [43] (*n* = 24)
ORR (95% CI)	15%	18.3% (14.0–23.4)	36% (26–46)	40.3% (28.5–53.0)	38.9%	13.6% (2.9–34.9)	54.2% (32.8–74.4)
DCR (95% CI)	55%	62.2%	71%	85.1% (74.3–92.6)	83.3%	68.2% (45.1–86.1)	91.7% (73.0–99.0)
PFS, months (95% CI)	3.7 (3.1–3.9)	3.0 (2.8–4.1)	7.4 (5.6–10.7)	9.7 (5.3–13.8)	7.2 (2.6–NE)	5.5 (1.9–7.4)	7.4 (3.7–NE)
OS, months (95% CI)	16.4 (13.9–18.4)	13.9 (11.6–16.0)	17.1 (13.8–NE)	20.4 (11.0–NE)	NR	12.7 (8.0–NE)	NR
DOR, months (M)	23.3 (3.1–34.5+)	13.8 (1.5–23.6)	NE (11.7–NE)	11.0 (5.6–11.0)	NA	5.5 (3.7–7.3)	NA

DCR, disease control rate; DOR, duration of response; NA, not available; NE; not evaluable; NR, not reached; ORR, objective response rate (RECIST 1.1); OS, overall survival; PFS, progression-fee survival. TKI, tyrosine kinase inhibitor.

**Table 2:** Phase III Clinical Trials of Advanced Stage HCC.

Target Population		Design	Trial Name	Result	Presentation	Publication	1st Author
**Advanced**	First line	1. Sorafenib vs. Sunitinib	SUN1170	Negative	ASCO 2011	JCO 2013	Cheng AL [47]
		2. Sorafenib ± Erlotinib	SEARCH	Negative	ESMO 2012	JCO 2015	Zhu AX [48]
		3. Sorafenib vs. Brivanib	BRISK-FL	Negative	AASLD 2012	JCO 2013	Johnson PJ [49]
		4. Sorafenib vs. Linifanib	LiGHT	Negative	ASCO-GI 2013	JCO 2015	Cainap C [50]
		5. Sorafenib ± Doxorubicin	CALGB 80802	Negative	ASCO-GI 2016		
		6. Sorafenib ±- HAIC	SILIUS	Negative	EASL 2016	Lancet GH 2018	Kudo M [51]
		7. Sorafenib ± Y90	SARAH	Negative	EASL 2017	Lancet-O 2017	Vilgrain V [52]
		8. Sorafenib ± Y90	SIRveNIB	Negative	ASCO 2017	JCO 2018	Chow PKH [53]
		9. Sorafenib vs. Lenvatinib	REFLECT	Positive	ASCO 2017	Lancet 2018	Kudo M [54]
		10. Sorafenib vs. Nivolumab	CheckMate-459	Negative	ESMO 2019		Yau T [34]
		11. Sorafenib ± Y90	SORAMIC	Negative	EASL 2018	J Hepatol 2019	Ricke J [55]
		12. Sorafenib vs. Atezolizumab + Bevacizumab	IMbrave150	Positive	ESMO-Asia 2019		Cheng AL [1]
		13. Sorafenib vs. Durvalumab + Tremelimumab vs. Durva	HIMALAYA	Ongoing			
		14. Sorafenib vs. Tislelizumab	Rationale301	Ongoing			
		15. Lenvatinib ± Pembrolizumab	LEAP002	Ongoing			
		16. Lenvatinib or Sorafenib vs. Nivolumab + Ipilimumab	CheckMate 9DW	Ongoing			
		17. Sorafenib vs. Atezolizumab + Cabozantinib	COSMIC-312	Ongoing			
	Second line	1. Brivanib vs. Placebo	BRISK-PS	Negative	EASL 2012	JCO 2013	Llovet JM [56]
		2. Everolimus vs. Placebo	EVOLVE-1	Negative	ASCO-GI 2014	JAMA 2014	Zhu AX [57]
		3. Ramucirumab vs. Placebo	REACH	Negative	ESMO 2014	Lancet-O 2015	Zhu AX [58]
		4. S-1 vs. Placebo	S-CUBE	Negative	ASCO 2015	Lancet GH 2017	Kudo M [59]
		5. ADI-PEG 20 vs. Placebo	NA	Negative	ASCO 2016	Ann Oncol 2018	Abou-Alfa GK [60]
		6. Regorafenib vs. Placebo	RESORCE	Positive	WCGC 2016	Lancet 2017	Bruix J [61]
		7. Tivantinib vs. Placebo	METIV-HCC	Negative	ASCO 2017	Lancet-O 2018	Rimassa L [62]
		8. Tivantinib vs. Placebo	JET-HCC	Negative	ESMO 2017		
		9. DT^#^ vs. Placebo	ReLive	Negative	ILCA 2017	Lancet Gastroenterol Hepatol	Merle P [63]
		10. Cabozantinib vs. Placebo	CELESTIAL	Positive	ASCO-GI 2018	NEJM 2018	Abou-Alfa G [64]
		11. Ramucirumab vs. Placebo	REACH-2	Positive	ASCO 2018	Lancet-O 2019	Zhu AX [65]
		12. Pembrolizumab vs. Placebo	KEYNOTE-240	Negative	ASCO 2019	JCO 2020	Finn RS [36]

Red: Positive trials, Blue: Ongoing trials, Black: Negative trials.
